# An Investigation of Self-Directed Learning Skills of Undergraduate Students

**DOI:** 10.3389/fpsyg.2018.02324

**Published:** 2018-11-23

**Authors:** İlkay Aşkin Tekkol, Melek Demirel

**Affiliations:** ^1^Department of Primary Education, Faculty of Education, Kastamonu University, Kastamonu, Turkey; ^2^Division of Lifelong Learning and Adult Education, Faculty of Education, Hacettepe University, Ankara, Turkey

**Keywords:** lifelong learning, undergradute students, self-direction, self-directed learning, self-directed learning skills

## Abstract

The aims of this study were to reveal university students' self-directed learning skills and determine whether these skills vary based on university type, gender, field of study, year of study, academic success, type of university entrance score, income level, and the desire to pursue a graduate degree. Also, this study explored the relationship between university students' self-directed learning skills and their lifelong learning tendencies. The study group of the survey comprised 2,600 first and fourth-year students from same departments of Hacettepe and Başkent Universities. The study collected its data by using “Self-Directed Learning Skills Scale” designed by Aşkin (2015). Moreover, Diker-Coşkun's “Lifelong Learning Tendencies Scale” was used to explore the relationship between university students' self-directed learning skills and their lifelong learning tendencies. The results revealed that university students' self-directed learning scores were above the median score of the scale. Self-directed learning skills were found not to vary based on university, year of study, income level. However, gender, field of study, university entrance score type, academic success and the desire to pursue a graduate degree made a significant difference on university students' self-directed learning skills. Finally, a moderate positive relationship was detected between self-directed learning skills and lifelong learning tendencies. In summary it can be said that, undergraduate students have self-directed learning skills and these skills are related to lifelong learning.

## Introduction

The ways of obtaining and using information have changed substantially in recent years as it has become accessible from multiple sources. This, in turn, has challenged the belief that information is unchangeable, as well as the belief that authorities have access to absolute and correct information. In addition, the view of learning as memorizing information in separate compartments gave way to a problem-oriented view based on conceiving, knowing and understanding (Aspin and Chapman, 2001). Therefore, the importance attached to memorization faded as conceiving the nature of knowledge and learning has changed, and learning how to learn, gained ground. Individuals who have learned how to learn can organize their own learning, transfer new information to larger contexts, overcome difficulties, and they are open to development and change, they possess self-confidence and awareness, they are willing to learn, they can use various learning strategies, and they know their own learning styles, interests and talents (Rawson, 2000; Giese, 2006; Fredriksson and Hoskins, 2007; Hofmann, 2008). Learning how to learn is among the fundamental skills of lifelong learning.

With lifelong learning, individuals can become aware of their own learning needs and they can decide how they want to reach knowledge. At the same time, they can understand the nature of knowledge instead of memorizing it. Lifelong learning enables individuals who seek self-development or further education to meet their learning needs independently and flexibly. With lifelong learning, these needs can be met anywhere both formally and informally (Aspin and Chapman, 2001). Lifelong learning deems continuity necessary between early learning experiences and work life. It is crucial to achieve such continuity between one school year and the next; one level and the next; early childhood education and elementary school; elementary and secondary school, secondary school and further levels of education and then work life (Organization for Economic Cooperation and Development, 1996). Lifelong learning may be associated with adult education; however, it cannot be limited to a certain age group as it lasts throughout a lifetime (Mocker and Spear, 1982). Lifelong learning focuses on knowledge and skills needed by everyone regardless of age. The literature reveals that lifelong learning covers various skills known as twenty first century skills and these skills are thought to be increasingly important in information societies. Self-directed learning skills are part of these skills. Literature shows that a close link exists between lifelong learning and self-direction. Greveson and Spencer (2005) claim that self-direction is a pre-requisite for lifelong learning, while Candy (1990) emphasizes that a mutual relationship exists between the two. According to Candy (1990), self-directed learning is a way of turning individuals into lifelong learners. On the other hand, one of the main aims of lifelong learning is to equip individuals with skills and competencies that enable them to learn by themselves. According to this belief, self-directed learning is both the meaning and the outcome of lifelong learning (Candy, 1990). Mocker and Spear (1982) on the other hand, assert that self-direction is a dimension of lifelong learning and facilitates it through formal and informal learning. According to Spencer and Jordan (1999), self-directed learning prepares individuals for lifelong learning. Brockett and Hiemstra (1991) state that self-direction needs to be considered with a perspective of (understanding of) lifelong learning. According to this, lifelong and self-directed learning are related concepts and they form basis to one another.

Also known as learning by oneself, self-directed learning in its largest sense refers to individuals ability to taking initiative to identify their own learning needs, their ability to determine their learning goals, their ability to define the sources they need in order to learn, their ability to choose/use appropriate learning strategies and evaluate learning outcomes with or without help from an outsider (Knowles, 1975). Self-directed learning is a process where individuals take primary charge of planning, continuing and evaluating their learning experiences (Merriam et al., 2007). In self-directed learning, the responsibility to learn shifts from an external source (teacher, etc.) to the individual. Control and active involvement of the learner in the learning process is crucial in this process (Boyer and Usinger, 2015; Grover, 2015). Self-directed learning includes the conceptualization, design, implementation and evaluation of learning guided by learners (Brookfield, 2009). It may be referred as a method of organizing learning which learners control the task of learning. In addition to these, self-directed learning may also be viewed as a target that learners strive to achieve. In order to achieve it, individuals take responsibility for their own learning and embrace individual autonomy and preferences (Kaufman, 2003). Self-directed learners have the following characteristics:

- They set clear goals for themselves.
- They shape their learning process in line with goals and plans.
- They monitor their own learning process.
- They evaluate the outcomes of their own learning.
- They are autonomous.
- They have self-motivation.
- They are open to learning.
- They are curious.
- They are willing to learn.
- They value learning.
- They have self-control.
- They take initiative to learn (Knowles, 1975; Knowles, 1977; Jennett, 1992 cited in Brockett and Hiemstra, 1991)

Self-directed learning enables individuals to improve their self-confidence, autonomy, motivation and lifelong learning skills (O'Shea, 2003). It turns learners into active participants in the learning process and encourages them to become deep learners (Spencer and Jordan, 1999). However, there are several competencies that self-directed learning requires. Knowles (1977) lists them as follows:

- The ability to enter into a close, respectful and learning-friendly relationship with learners
- The ability to establish an environment which is physically and psychologically comfortable, open to interaction, based on cooperation, open and secure
- The ability to take responsibility for determining one's own learning needs
- The ability to set goals
- The ability to plan, implement and evaluate learning activities
- The ability to help learners to self-direct their learning
- The ability to be a facilitator and a source
- The ability to effectively use small group processes
- The ability to evaluate learning processes and outcomes (Knowles, 1977 cited in Kasworm, 1983).

Studies on self-directed learning show that the Turkish literature refers to the concept in a number of different ways. While some studies refer to it as learning by oneself, others use the term self-regulated learning or self-directed learning. The majority of self-directed learning studies seems to have been conducted with university students. Fewer international and national studies have taken secondary and high school students as their focus. Both descriptive and experimental studies have been conducted. Their design has largely been quantitative, with a few qualitative studies. Studies in Turkey have mostly adapted and used Fisher et al.'s (2001) “Self-Directed Learning Readiness Scale.” In addition, Guglielmino's “Self-Directed Learning Readiness Scale” was also preferred by some researchers. In addition, there are also scales developed by researchers, albeit in small numbers. These scales mostly aimed at revealing department related or field of study-related information (such as scales for field of science, scales to be used with teacher candidates, in laboratories, etc.), and did not used for measuring students' general self-directed learning skills.

In international literature a lot of studies have been about self-directed learning and there are many scale development studies have been conducted measuring self-directed learning skills (Guglielmino, 1977; Oddi, 1984; Fisher et al., 2001; Williamson, 2007; Hendry and Ginns, 2009; Stockdale and Brockett, 2010; Shen et al., 2014; Cadorin et al., 2017; Lopes and Cunha, 2017). Also in some studies there have been recommended models for development of university students (Aly et al., 2003; Mamary and Charles, 2003; Beach, 2017; Sawatsky et al., 2017). Most of the participants in the studies are university students and adults.

The results of previous studies have shown that self-directed learning is linked with upper level thinking skills such as creativity, problem solution and critical thinking. In addition, certain studies have shown that academic success is also closely linked to self-directed learning. Regarding the variable of gender, some studies found no significant effect while others found one in favor of female students. Other findings showed that students desiring to pursue a graduate degree have higher self-directional learning skills and these skills are not affected by variables such as school type, year of study, race, or income level.

### Purpose and importance of the study

This study aims to determine the self-directed learning skills of university students and to reveal whether these skills vary based on university type, gender, field of study, year of study, academic success, type of university entrance score, income level, and the desire to pursue a graduate degree. These variables were determined from the findings of the researches on the subjects are self-regulated learning, lifelong learning tendencies, lifelong learning competencies, learning approaches, etc., which are related to self-directed learning. In addition, in the field of lifelong learning competences, self-determination, self-evaluation, willingness to learn, determination of appropriate learning strategies, learning to learn, and so on. It is also aimed to reveal the relationship between self-directed learning skills and lifelong learning tendencies. Because in literature self-directed learning is considered as a dimension of lifelong learning.

Self-directed learners are individuals who set themselves clear goals, act upon plans, take initiative, are open to learning, they are motivated, self-confident and self-controlled. In our day when information increases exponentially, these qualities are required from up-to-date individuals. After all, people who can direct their own learning have acquired ways of reaching information, can think at higher levels and organize their own learning. In short, they are individuals who have mastered how to learn. Having these qualities would enable university students self-development both personally and professionally after university life, They have a will to learn, they are open to sustained learning and they tend to sustain learning; in short, they become lifelong learners. Therefore, it is important to identify university students' self-directed learning skills. The following constitute the subproblems of the study:

- Do Hacettepe and Başkent University students' self-directed learning skills differ significantly based on university type?
- Do Hacettepe and Başkent University students' self-directed learning skills differ significantly based on gender?
- Do Hacettepe and Başkent University students' self-directed learning skills differ significantly based on field of study?
- Do Hacettepe and Başkent University students' self-directed learning skills differ significantly based on year of study?
- Do Hacettepe and Başkent University students' self-directed learning skills differ significantly based on academic success?
- Do Hacettepe and Başkent University students' self-directed learning skills differ significantly based on university entrance score type?
- Do Hacettepe and Başkent University students' self-directed learning skills differ significantly based on desire to pursue a graduate degree?
- Do Hacettepe and Başkent University students' self-directed learning skills differ significantly based on income level?
- Is there a significant relationship between Hacettepe and Başkent University students' self-directed learning skills and lifelong learning tendencies?

### Limitations of the study

This study is limited with;

- Hacettepe and Başkent Universities in Ankara,
- Departments that are same in the determined universities (Computer Engineering, Electrical and Electronic Engineering, Industrial Engineering, Medicine, Dentistry, Nursing, Social Services, Nutrition and Dietetics, Physiotherapy and Rehabilitation, Business Administration, Economics, Psychology, Turkish Language and Literature, Early Childhood Education, Elementary Education, Elementary Mathematics Education, English Language Education, and Psychological Counseling and Guidance, Interior Design and Environment Design, Graphic Design, Musical/Performance Arts)
- First and fourth year students studying in determined departments of Hacettepe and Başkent universities,
- Self-directed learning skills scale.

## Material and methods

This study aims to identify university students' self-directed learning skills and to explore the relationship between these skills and university type, gender, field of study, year of study, level of academic success, university entrance score type, students' desire to pursue a graduate degree and income level. In other words, university students' self-directed learning skills was investigated. Therefore, the study used the survey method (descriptive method).

### Study group

This study conducted at two universities in the province of Ankara. One of them is state and one of them is private. State university is Hacettepe University and private university is Başkent University. The aim is to find out whether there is a significant difference between the state and private universities in terms of self-directed learning skills. The reason for opting for Başkent University to conduct the research, among other private universities is that it is the private university which shows the most similarity with Hacettepe University in terms of the same faculties and departments (e.g., faculty of education).

The study group comprised 2,600 first and fourth year students from the Engineering, Medicine, Dentistry, Fine Arts, Nursing, Health Sciences, Administrative Sciences, Education, and Science, and Letters Faculties and the State Conservatory of Hacettepe and Başkent Universities located in Ankara. The departments that exist in both universities were included in the study: Computer Engineering, Electrical and Electronic Engineering, Industrial Engineering, Medicine, Dentistry, Nursing, Social Services, Nutrition and Dietetics, Physiotherapy and Rehabilitation, Business Administration, Economics, Psychology, Turkish Language and Literature, Early Childhood Education, Elementary Education, Elementary Mathematics Education, English Language Education, and Psychological Counseling and Guidance, Interior Design and Environment Design, Graphic Design, Musical/Performance Arts. The distribution of the study group is shown in Table 1.

**Table 1:** The distribution of the study group according to university, fields and departments.

**Field of study**	**Departments**	**Students**								**Total**	**Extreme values removed**
		**Hacettepe**				**Başkent**					
		**1st year**		**4th year**		**1st year**		**4th year**			
		***f***	**%**	***f***	**%**	***f***	**%**	***f***	**%**		
Natural Sciences	Computer Eng. Electrical and Electronics Eng. Industrial Eng. Elementary Mathematics Ed.	167	37.11	97	21.56	80	17.78	106	23.56	450	444
Fine arts	Graphic Design Internal Design and Environment Design Musical/ Performance Arts	51	35.66	29	20.28	42	29.37	21	14.69	143	142
Health sciences	Nutrition and Dietetics Dentistry Physiotherapy and Rehab. Nursing Social Services Medicine	585	52.23	209	18.66	181	16.16	145	12.95	1120	1105
Social sciences	Turkish language and Literature Psychology Early Childhood Ed. Elementary Ed. English Language Ed. Guidance and Psychological Counseling, Economics, Business Administration	255	27.72	348	37.83	173	18.80	144	15.65	920	909
Total		1058		683		476		416		2633	2600

According to Table 1, there were 450 Natural Sciences students, 143 Fine Arts students, 1,120 Health Sciences students, and 920 Social Sciences students. Data from a total of 2,633 students were analyzed and extreme values were removed. As a result, the study group consisted of 2,600 individuals.

### Data collection tools

Data for the study were collected by the “Self-Directed Learning Skills Scale,” “Lifelong Learning Tendencies Scale” and a personal information form. The personal information form included questions about students' gender, field of study, year of study, academic success, university entrance score type, income level, and the desire to pursue a graduate degree.

### Self-directed learning skills scale

“Self-Directed Learning Skills Scale” developed by Aşkin (2015) in order to reveal university students' self-directed learning skills. Content and construct validity was tested to ensure the validity of the scale. 53-item trial version was implemented on 753 students attending Hacettepe and Başkent Universities during the fall term of 2014–2015 academic year. According to the exploratory factor analysis, four factors with an eigenvalue above 1 were emerged. Confirmatory factor analysis was performed on the data from the 2,600 students in the study group following the real implementation in order to test the construct validity of the scale. RMSEA value calculated as 0.069. An RMSEA value below 0.08 shows “acceptable fit” (1992). A chi square value was found as 2424.14. The value obtained by dividing the chi square value by the degree of freedom was 13.26. This value being five or below shows good fit (Çokluk et al., 2012). And the fit indices of the scale were GFI 0.92; AGFI 0.89; CFI 0.96; NFI 0.96; NNFI 0.96, and SRMR was below 0.05. These were shown to range between good and acceptable values (Schermelleh-Engel and Moosbrugger, 2003). According to this, the 21-item and four-factor structure of the “Self-Directed Learning Skills Scale” was confirmed as a model (Aşkin, 2015).

The Cronbach Alpha internal consistency coefficient was used to explore the reliability of the “Self-Directed Learning Skills Scale.” The reliability coefficients of the dimensions of the scale were calculated: The subdimensions of motivationwas 0.826, self-control was 0.799, self-monitoring was 0.768, and self-confidence was 0.690. The Cronbach Alpha internal consistency coefficient for the entire scale (21 items) was 0.895, suggesting that the final version of the scale had high reliability (Aşkin, 2015).

### Lifelong learning tendencies scale

Designed by Diker-Coşkun (2009), the “Lifelong Learning Tendencies Scale” is a 6-point Likert type scale aiming to measure university students' lifelong learning tendencies. The 74-item trial version of the scale was shaped in line with expert views, its correlation was measured with the “Curiosity Index” Scale, known to measure the same construct. This value was 0.76. The trial version was implemented on 642 students attending different faculties and departments of seven Turkish universities with different characteristics. The data obtained were subjected to exploratory factor analysis and the scale was found to have four subdimensions: motivation, perseverance, inability to regulate learning, lack of curiosity. The total reliability for the final form of the 27-item scale was 0.89. The reliability of the “Lifelong Learning Tendencies Scale” in this study was 0.917 (Diker-Coşkun, 2009).

These scales were chosen because they were valid and reliable scales measuring self-directed learning skills and lifelong learning skills of undergraduate students.

### Data collection procedures

The data of the research were gathered by the researchers from the students who were educated in the first and fourth grades of the determined sections of Hacettepe and Başkent Universities with the “Self-Directed Learning Skills” and “Lifelong Learning Tendencies” Scales. Research ethics committee approval was obtained from the Ethics Committee of Hacettepe University and Başkent University Rectorate before data collection process. Before collecting data, written informed consent was obtained from all participants.

### Data analysis

Out of the 2,704 data form collected, 91 that were not marked properly were excluded, and data from 2,633 students were computed. The analyses were conducted on SPSS 22 package. Following the extreme value analysis performed here, 33 more forms were excluded and a total of total 2,600 student forms were included in the analyses. Data were analyzed by using descriptive statistics, *t*-test, one way analysis of variance (ANOVA) and Pearson Moments Correlation Coefficient.

## Results

### Descriptive statistics relating to self-directed learning skills scores

The distribution of university students' self-directed learning skills are shown in Table 2.

**Table 2:** Arithmetic means and standard deviations of students' self-directed learning skills scores.

	***n***	**Lowest**	**Highest**	**$\bar{\text{X}}$**	***ss***
Scale total	2,600	44	105	84.02	10.27
1st Subdimension: Motivation	2,600	14	35	29.74	3.90
2nd Subdimension: Self-Control	2,600	7	25	17.91	3.40
3rd Subdimension: Self-Monitoring	2,600	8	25	19.50	2.91
4th Subdimension: Self-Confidence	2,600	7	20	16.87	2.21

According to Table 2, the lowest score obtained from the Self-Directed Learning Scale was 44, and the highest score was 105. The mean of the scale was measured as 84.02. The highest possible score from the scale was 105 (21 × 5), median was 63 (21 × 3) and the lowest one was 21 (21 × 1). Students' self-directed learning skills arithmetic means were above the median of the scale. Subdimension statistics show that the mean in the motivation subdimension was 29.74; in self-monitoring was 19.50; in self-control was 17.91 and in self-confidence was 16.87.

Computations showed that the highest, lowest and median scores from the subdimensions were 35, 7, and 21 in motivation; 25, 5, and 15 in self-control; 25, 5, and 15 in self-monitoring; and 20, 4, and 12 in self-confidence. The mean scores of the subdimensions were higher than their own medians. The distribution of university students' responses can be seen in Table 3.

**Table 3:** Distribution of responses to the “self-directed learning skills scale.”

**Item No**	**Always**		**Generally**		**Sometimes**		**Rarely**		**Never**		**$\bar{X}$**
	***f***	**%**	***f***	**%**	***f***	**%**	***f***	**%**	***f***	**%**
1	819	31.5	1,451	55.8	311	12	19	0.7	0	0	4.18
2	957	31.5	1,215	46.7	398	15.3	30	1.2	0	0	4.19
3	688	26.5	1,231	47.3	611	23.5	63	2.4	7	0.3	3.97
*4*	*1,352*	*52*	*1,012*	*38.9*	*203*	*7.8*	*33*	*1.3*	*0*	*0*	*4.42*
5	1,259	48.4	898	34.5	354	13.6	69	2.7	20	0.8	4.27
6	1,271	48.9	944	36.3	297	11.4	48	1.8	40	1.5	4.29
7	625	24	1,185	45.6	648	24.9	130	5	12	0.5	3.88
8	1,309	50.3	948	36.5	290	11.2	53	2	0	0	4.35
*9*	*348*	*13.4*	*866*	*33.3*	*1028*	*39.5*	*326*	*12.5*	*32*	*1.2*	*3.45*
10	465	17.9	992	38.2	855	32.9	247	9.5	41	1.6	3.61
11	1,006	38.7	1.063	40.9	416	16	103	4	12	0.5	4.13
12	404	15.5	999	38.4	857	33	293	11.3	47	1.8	3.55
13	1,125	43.3	924	35.5	477	18.3	61	2.3	13	0.5	4.19
*14*	*326*	*12.5*	*828*	*31.8*	*1009*	*38.8*	*385*	*14.8*	*52*	*2*	*3.38*
15	1,006	38.7	1,056	40.6	429	16.5	92	3.5	17	0.7	4.13
16	682	26.2	977	37.6	729	28	197	7.6	15	0.6	3.81
17	1,200	46.2	828	31.8	371	14.3	144	5.5	57	2.2	4.14
18	594	22.8	1,189	457	676	26	127	4.9	14	0.5	3.85
19	1,248	48	1,029	39.6	263	10.1	60	2.3	0	0	4.33
20	482	18.5	1,113	42.8	819	31.5	163	6.3	23	0.9	3.72
21	1,056	40.6	1,025	39.4	424	16.3	70	2.7	25	1	4.16

*Italic item numbers indicate highest and lowest arithmetic means*.

As presented in Table 3, university students' self-directed learning skills means were generally high (Mean scores are 3.41 and above). The item with the highest mean score was “I am open to learning” (4; 4.42) and the one with the lowest mean was (14) “I systematically monitor my learning process” (3.38). The item “I review my learning process regularly” (9) had a lower mean score than others (3.45).

### Self-directed learning skills and university type

Table 4 shows the *t*-test analysis results to reveal whether self-directed learning skills vary by university type.

**Table 4:** Independent groups *t*-test statistics of self-directed learning skills by university type.

**University**	***n***	**$\bar{X}$**	***ss***	***Sd***	***t***	***p***
Hacettepe	1,714	84.018	10.18	2,598	−0.012	0.990
Başkent	886	84.022	10.45	

Table 4 shows that Hacettepe University students' self-directed learning skills mean score was 84.018, and that of Başkent University students was 84.022. Independent groups *t*-test was performed to explore whether a significant difference existed between students' self-directed learning skills and the type of their university. According to *t*-test results, university type did not cause an effect on self-directed learning skills (*p* > 0.05).

### Self-directed learning skills and gender

The relationship between Hacettepe and Başkent University students' self-directed learning skills and gender are given in Table 5.

**Table 5:** Independent groups *t*-test statistics of self-directed learning skills by gender.

**Gender**	***n***	**$\bar{X}$**	***ss***	***sd***	***t***	***p***
Female	1,894	84.71	9.98	2,598	5,618	0.000
Male	706	82.18	10.81	

According to Table 5, female students' self-directed learning skills mean score was 84.71, and that of male students was 82.18. The independent groups *t*-test conducted to reveal whether there was a significant difference between university students' self-directed learning skills and gender showed that a significant difference existed between gender and self-directed learning skills (*p* < 0.05) in favor of female students.

### Self-directed learning skills and field of study

The relationships between university students' self-directed learning skills and their field of study are given in Table 6.

**Table 6:** Arithmetic means and standard deviations of self-directed learning skills by field of study.

**Field of study**	***n***	**$\bar{X}$**	***ss***
Natural Sciences	444	81.85	10.19
Fine Arts	142	85.70	10.72
Health Sciences	1,105	84.15	10.15
Social Sciences	909	84.66	10.24
Total	2,600	84.02	10.27

As shown in Table 6, the lowest self-directed learning skills scores were obtained by students in the Natural Sciences field of study (81.85); followed by Health Sciences (84.15) and Social Sciences (84.66). On the other hand, the highest mean score belonged to the Fine Arts field of study (85.70). The ANOVA results showed a significant difference between university students' self-directed learning skills and their field of study (*p* < 0.05). The Bonferroni test was run to explore the fields of study between which the difference existed. The analysis results are given in Table 7.

**Table 7:** Group comparisons of self-directed learning skills by field of study.

**Groups**	**Difference in mean score**	
Natural Sciences	Fine Arts	−3.84808(^*^)
	Health Sciences	−2.29932(^*^)
	Social Sciences	−2.81097(^*^)
Fine Arts	Natural Sciences	3.84808(^*^)
	Health Sciences
	Social Sciences
Health Sciences	Natural Sciences	2.29932(^*^)
	Fine Arts
	Social Sciences
Social Sciences	Natural Sciences	2.81097(^*^)
	Fine Arts
	Health Sciences

* *means there is significant difference between groups*.

According to Table 7, there were significant differences between Fine Arts, Health Sciences and Social Sciences students' self-directed learning skills mean scores. The difference was in favor of Health Sciences, Social Sciences and Fine Arts. Their self-directed learning skills mean scores were significantly higher than those of Natural Sciences students.

### Self-directed learning skills and year of study

Below in Table 8 results belongs to the independent groups *t*-test results of university students' self-directed learning skills and years of study.

**Table 8:** Independent groups *t*-test statistics of self-directed learning skills by year of study.

**Year of study**	***n***	**$\bar{X}$**	***ss***	***sd***	***t***	***p***
1st Year	1,513	84	10.32	2,598	0.443	0.895
4th Year	1,087	84.05	10.20			

Table 8 shows that first-year students' self-directed learning skills mean score was 84; while that of fourth-year students was 84.05. Independent groups *t*-test was also performed to investigate the presence of a significant difference between university students' year of study and self-directed learning skills. The analysis revealed that year of study did not cause a difference in self-directed learning skills (*p* > 0.05).

### Self-directed learning skills and academic success

Table 9 shows the relationships between Hacettepe and Başkent University students' self-directed learning skills and their academic success.

**Table 9:** Arithmetic means and standard deviations of self-directed learning skills by academic success level.

**Academic success level**	***n***	**$\bar{X}$**	***ss***
0–0.99	34	77.26	12.13
1.00–1.49	50	81.88	12.04
1.50–1.99	176	82.78	9.84
2.00–2.49	424	82.01	10.84
2.50–2.99	828	84	9.84
3.00–3.49	880	84.76	10.18
3.50 and above	208	87.74	9.14
Total	2600	84.02	10.27

Table 9 reveals that the highest self-directed learning mean was obtained by students with a GPA of 3.5 and above (87.74). This group was followed by those with a GPA of 3.00–3.49 (84.76), and then with 2.50–2.99 (84); 1.50–1.99 (82.78); 2.00–2.49 (82.01); 1.00–1.49 (81.88). The lowest mean was in the 0–0.99 interval (77.26). ANOVA was also conducted to reveal whether academic success levels differed significantly, and a significant difference was found between university students' self-directed learning skills and academic success (*p* < 0.05). The Bonferroni test was conducted to find where the difference stemmed from. The results can be seen in Table 10.

**Table 10:** Group comparisons of self-directed learning skills by academic success.

**Groups**	**Differences in mean scores**	
0–0.99	0–0.99 1.00–1.49 1.50–1.99 2.00–2.49 2.50–2.99 3.00–3.49 3.50 and above	−6.73771 (^*^) −7.49098 (^*^) −10.47087(^*^)
1.00–1.49	0-0.99 1.00–1.49 1.50–1.99 2.00–2.49 2.50–2.99 3.00–3.49 3.50 and above	−5.85558(^*^)
1.50–1.99	0–0.99 1.00–1.49 2.00–2.49 2.50–2.99 3.00–3.49 3.50 and above	−4.95149(^*^)
2.00–2.49	0–0.99 1.00–1.49 1.50–1.99 2.50–2.99 3.00–3.49 3.50 and above	−1.99534(^*^) −2.74861(^*^) −5.72850(^*^)
2.50–2.99	0–0.99 1.00–1.49 1.50–1.99 2.00–2.49 3.00–3.49 3.50 and above	6.73771(^*^) 1.99534(^*^) −3.73316(^*^)
3.00–3.49	0–0.99 1.00–1.49 1.50–1.99 2.00–2.49 2.50–2.99 3.50 and above	7.49098(^*^) 2.74861(^*^) −2.97990(^*^)
3.50 and above	0–0.99 1.00–1.49 1.50–1.99 2.00–2.49 2.50–2.99 3.00–3.49	10.47087(^*^) 5.85558(^*^) 4.95149(^*^) 5.72850(^*^) 3.73316(^*^) 2.97990(^*^)

* *means there is significant difference between groups*.

Table 10 shows significant differences between self-directed learning skills at different levels of academic success. Individuals with a GPA of 3.5 and above differed significantly from all others with their self-directed learning skills scores of 0–0.99. On the other hand, those with a GPA of 2.50–2.99 and 3.00–3.49 also differed significantly from those with a GPA of 2.00–2.49.

### Self-directed learning skills and university entrance score type

The analysis of Hacettepe and Başkent University students' self-directed learning skills according to their university entrance score type is shown in Table 11.

**Table 11:** Arithmetic means and standard deviations of self-directed learning skills by university entrance score type.

**University entrance score type**	***n***	**$\bar{X}$**	***ss***
Mathematics-Science (MS)	1,426	83.34	10.21
Turkish-Mathematics (TM)	895	84.36	10.15
Turkish-Social Studies (TS)	104	87.66	9.44
Language	105	83.98	11.16
Talent Score	70	88.14	10.73
Total	2,600	84.02	10.27

Table 11 shows university students' self-directed learning skills and university entrance score types. The highest mean score belongs to students who entered university with a talent score (88.14). They are followed by those who entered university with a Turkish-Social Studies (TS) score (87.66); Turkish-Mathematics (TM) score (84.36); and Language score (83.98). Students with the lowest score were those who entered university with the Mathematics-Science score type (83.34). ANOVA results revealed a significant difference between university students' self-directed learning skills according to their university entrance score types. The Bonferroni test conducted to identify the source of the difference yielded the following results included in Table 12.

**Table 12:** Group comparisons of self-directed learning skills by university entrance score type.

**Groups**	**Difference in mean scores**	
Mathematics-Science (MS)	Turkish-Mathematics (TM) Turkish-Social Studies (TS) Language Talent Score	−4.32054 (^*^) −4.79994 (^*^)
Turkish-Mathematics (TM)	Mathematics-Science (MS) Turkish-Social Studies (TS) Language Talent Score	−3.30815 (^*^) −3.78755 (^*^)
Turkish-Social Studies (TS)	Mathematics-Science (MS) Turkish-Mathematics (TM) Language Talent Score	4.32054 (^*^) 3.30815 (^*^)
Language	Mathematics-Science (MS) Turkish-Mathematics (TM) Turkish-Social Studies (TS) Talent Score
Talent Score	Mathematics-Science (MS) Turkish-Mathematics (TM) Turkish-Social Studies (TS) Language	4.79994 (^*^) 3.78755 (^*^)

* *means there is significant difference between groups*.

According to Table 12, there are significant differences between Mathematics-Science (MS), Turkish-Mathematics (TM), Turkish-Social Studies (TS) and talent scores. The difference was in favor of Turkish-Social Studies (TS) and talent scores. Turkish-Social Studies (TS) and talent scores were significantly higher than Mathematics-Science (MS) and Turkish-Mathematics (TM) scores.

### Self-directed learning skills and the will to pursue a graduate degree

The results of the analysis aboutrelationship between Hacettepe and Başkent University students' self-directed learning skills and their desire to pursue a graduate degree are given in Table 13.

**Table 13:** Independent groups *t*-test statistics of self-directed learning skills by the will to pursue a graduate degree.

**Will to pursue graduate education**	***n***	**$\bar{X}$**	***ss***	***sd***	***t***	***p***
Yes	1,947	84.87	10.04	2,598	7,406	0.000
No	653	81.47	10.52	

Table 13 shows that the self-directed learning mean score of students who desired to pursue graduate education was 84.87 and that of others was 81.47. The *t*-test results show a significant relationship between desire to pursue a graduate degree and self-directed learning skills (*p* < 0.05). The difference was in favor of those who desired to pursue graduate education.

### Self-directed learning skills and income level

Table 14 shows the results of the analysis performed to reveal whether university students' self-directed learning skills differed significantly with respect to income level.

**Table 14:** Arithmetic means and standard deviations of self-directed learning skills by income level.

**Monthly Income**	**n**	**$\bar{X}$**	**ss**
0–999	176	87.76	9.39
1,000–1,999	496	84.29	10.45
2,000–2,999	572	84.98	9.46
3,000–3,999	468	83.95	10.67
4,000–4,999	266	83.08	10.51
5,000 and above	622	84	10.66
Total	2,600	84.02	10.27

As presented in Table 14, the income group with the highest self-directed learning skills mean score was 0–999 (84.98). This was followed by the groups with the income levels 2,000–2,999 (84.98); 1,000–1,999 (84.29); 3,000–3,999 (83.95), and 5,000 and above. The lowest group was 4,000–4,999 (83.08). According to ANOVA results, no significant difference existed between self-directed learning skills (*p* > 0.05) by the level of income.

### Relationship between self-directed learning skills and lifelong learning tendencies

The results of the analysis conducted to show the relationship between university students' self-directed learning skills and their lifelong learning tendencies are presented in Table 15.

**Table 15:** The relationship between university students' self-directed learning skills and lifelong learning tendencies.

		**Self-directed** **learning**	**Lifelong learning tendencies**
Self-directed learning	Pearson	1	0.511^*^
	*p*		0.000
	*n*	2,600	2,600
Lifelong learning tendencies	Pearson	0.511^*^	1
	*p*	0.000
	*n*	2,600	2,600

* *means there is significant relationship between self-directed learning and lifelong learning tendencies*.

In order to study the relationship between university students' self-directed learning skills and lifelong learning tendencies, the Pearson's Product Moment Correlation Coefficient was calculated. Table 15 shows a significant relationship between university students' self-directed learning skills and lifelong learning tendencies. This was a moderate and positive relationship (*p* < 000; 0.511).

## Discussion

The aims of this study were to examine university students' self-directed learning skills and reveal how these skills vary based on type of university, gender, area of study, year of study, academic success, university entrance score type, the desire to pursue a graduate degree and income level. In addition, this study also examined the relationship between university students' self-directed learning skills and their lifelong learning tendencies. The results showed that university students' self-directed learning skills were above the median of the scale. The scores that students obtained from the subdimensions of the scale, namely motivation, self-monitoring, self-control and self-confidence, were also above their medians. The highest average was obtained from the item “I am an individual open to learning”. According to this, university students define themselves as individuals who are open to learning. “Openness” is among the basic qualities of individuals who are self-directed learners (Oddi, 1984). Studies on self-directed learning skills also report that “openness” is the most important trait related to self-directed learning (Cazan and Schiopca, 2014). It may therefore be claimed that students of Hacettepe and Başkent Universities possess the quality of “openness” which is among the fundamental qualities of self-directed learning skills. The item with the lowest average was “I monitor my learning process systematically”. The item “I review my learning process regularly” also had a low average. Students' monitorization of their own learning has an important place in self-directed learning (Knowles, 1975) because it is critical in this type of learning for learners to monitor their learning in line with their own needs and goals, to identify wrong or deficient learning and to use new strategies accordingly. Kiliç and Sökmen (2012) studied teacher candidates' self-directed learning skills and concluded that their lowest scoring dimension was “self-control”. Even though Hacettepe and Başkent University students' self-directed learning skills were generally high, their scores from the “monitoring the learning process” and “regularly reviewing the learning process” skills in the self-control dimension were lower.

The students may have obtained higher self-directed learning skills average scores than the median may be attributed to the fact that the study group included university students. After all, self-directed learning is a concept associated with adult education, and it involves individuals decision of their own needs and goals and their ability to shape their learning accordingly. To be admitted to a university, students need a certain achievement level and CGPA. Therefore, their success depends on their managing, monitoring, evaluating skills and, when necessary, their ability to reorganize their own learning. According to Edmondson et al. (2012), students who use self-directed learning effectively are more successful. Ilhan-Beyaztaş (2014) states that successful students recommended the following to become effective learners: identifying goals and planning to achieve them; organizing study environments in line with their goals, doing research, accepting help from others when necessary, and monitoring their own learning. These recommendations overlap with the characteristics of self-directed learners. According to this, self-directed learning skills are expected from individuals of a certain success level admitted at universities. These corroborate the results of the study. In literature there were studies about undergraduate students and different occupational groups self-directed learning skills were found above the average (Premkumar et al., 2014; Cook et al., 2017; Swart, 2018). Also, previous studies focusing on self-regulatory learning skills, which are similar to self-directed learning skills (Yen et al., 2005; Turan, 2009; Çelik, 2012), also showed that students seem to possess moderate and high self-regulatory learning skills.

No significant difference was detected between Hacettepe and Başkent University students' self-directed learning skills and university type. The reason for this may have been that the self-directed learning skills of the students from the two institutions were largely similar. Even though the two universities have differences (state vs. private university), their students are believed to have similar characteristics regarding self-directed learning skills. Also, the two institutions are academically similar, thus following similar practices to develop their students' self-directed learning skills. Previously, Turner's study Turner (2007) with students from different high schools also showed that their self-directed learning readiness levels did not differ significantly.

Analysis of self-directed learning skills with respect to gender showed that a significant difference existed between female and male students' self-directed learning skills. The results showed that female students had significantly higher self-directed learning skills than male students. This can be attributed to the differences between female and male students regarding issues that may be considered indicators of self-directed learning (using learning strategies, motivation for learning, time management, planning, etc.). Aydemir (2007) concluded in her study that female students used learning strategies more often in the English course and did more inner loading regarding their failures. Saban (2008) studied teacher candidates and showed that female students have higher cognitive awareness and motivation levels than male students. Demirtaş and Özer (2007) found that female teacher candidates have more effective time management. Karasakaloglu and Saracaloglu (2009), on the other hand, state that female students have higher academic self design in the field of Turkish than male students. Higher self-directed learning skills in female students than males may be associated with the fact that the former have higher cognitive and affective characteristics, which are critical for applying self-directed learning. The literature also includes other studies that show higher self-directed learning skills among females (Guglielmino et al., 1987; Hutto, 2009; Slater et al., 2017; Swart, 2018). Similarly, studies on the relationship between lifelong learning tendencies and gender also showed that lifelong learning tendencies of females were significantly higher than those of males (Diker-Coşkun, 2009; Izci and Koç, 2012; Erdogan, 2014).

Another variable explored in the study was field of study. When students' self-directed learning skills scores were analyzed with respect to field of study, the lowest score belonged to Natural Sciences students. They were followed by Health Sciences and Social Sciences students. The field with the highest self-directed learning skills scores was Fine Arts. University students' self-directed learning skills differed significantly according to their fields of study. It was seen that Fine Arts, Social Sciences and Health Sciences students had significantly higher self-directed learning skills than Natural Sciences students. The highest self-directed learning skills mean score belonging to Fine Arts students may be attributed to the high creativity of students in this faculty. According to San (1983), one of the aims of art education is “to train individuals who are open to learning and creative in all walks of life. In other words, the purpose of art education is to become independent and to have productive thought and behaviors, to be entrepreneurial and to engage in creative activity.” Therefore, individuals who receive art education are naturally creative. Aral (1999) also concluded that art students have significantly higher creativity than others. The literature also documents the relationship between self-directed learning and creativity (Guglielmino et al., 1987; Cox, 2002; Edmondson et al., 2012). The tendency of Fine Arts and Social Sciences students for continuous learning may be the reason why their self-directed learning skills scores are higher than those of Natural Sciences students. Duman's (2004) study documents this. In this study, Duman (2004) explored the motivational orientations of students from Schools of Social Sciences and Natural Sciences. While Social Sciences students had a higher desire to learn and owned learning orientation which includes a strong desire to know, Natural Sciences students owned goal orientation which includes achieving clearly identified goals and repeating learning, but not a clear tendency for continuous learning. Previous studies also found higher lifelong learning tendencies scores, which are closely linked to self-directed learning, among Fine Arts and Social Sciences students than Natural Sciences (Diker-Coşkun, 2009; Kozikoglu, 2014; Yaman, 2014).

The study also explored whether students' self-directed learning skills varied based on year of study. First- and fourth-year students' self-directed learning skills scores were very close. The analyses showed no significant difference between Hacettepe and Başkent University students' self-directed learning skills according to the year of study. The reason behind self-directed learning does not depend on year of study may be due to the fact that self-directed learning is not completely dependent on formal education, but individuals' own characteristics. Self-directed learning is a skill that can also be developed informally. Gibbons and Phillips (1982) argue that self-directed learning occurs outside of formal education institutions. Gibbons and Phillips (1982) state that while self-directed learning skills are teachable and feasible for schools, they cannot be acquired by following a curriculum, it can be achieved only by allowing individuals to choose what they want to learn. This may be the reason why there was no correlation appeared between year of study in a formal education institution and self-directed learning skills. Previous studies in the literature also corroborate this finding (Salas, 2010; Kiliç and Sökmen, 2012; Acar, 2014; Sarmasoglu and Görgülü, 2014). Similarly, studies which explored the existence of a relationship between year of study and lifelong learning tendencies, which are related to self-directed learning skills, also concluded that there was no significant difference existed between the two (Diker-Coşkun, 2009; Erdogan, 2014).

The analysis of the difference between academic success and self-directed learning skills showed significant differences of self-directed learning skills according to the level of academic success. Students with higher academic success were found to have significantly higher self-directed learning skills. Self-directed learners are individuals who can identify their own learning needs, also they can utilize different learning strategies, methods and techniques, manage their learning processes, plan their time effectively, evaluate their learning outcomes, and identify and amend their learning deficiencies. These skills are also related to academic success. Demirtaş and Özer (2007) showed a significant relationship between time management and academic success. Subaşi (2000) showed that effective study methods such as identifying priorities, planning time, utilizing different learning strategies and managing studies increase academic success. These findings explain the high levels of academic success among individuals capable of self-directed learning. Studies cited in the literature also document a relationship between self-directed learning skills and academic success (Haggerty, 2000; Hall, 2011; Edmondson et al., 2012; Avdal, 2013; Karataş, 2013; Acar, 2014; Cazan and Schiopca, 2014; Sarmasoglu and Görgülü, 2014). Similarly, lifelong learning, which is related to self-regulation, has also been associated in the literature with academic success (Diker-Coşkun, 2009). Also, there are studies that show that individuals with academic success also have high self-organization skills (Üredi and Üredi, 2005; Turan, 2009).

In this study which explored university students' self-directed learning skills with respect to university entrance score type. The highest score was found to belong to students who entered university with a talent score. They were followed by students who enter university with Turkish-Social Studies (TS), Turkish-Mathematics (TM) and language scores. The lowest mean scores belonged to those who entered university with Mathematics-Science (MS) score type. There were significant differences of self-directed learning skills according to the types of entrance score. The self-directed learning skills of students who entered university with Talent and Turkish-Social Studies (TS) scores were found to be significantly higher than those who entered university with Turkish-Mathematics (TM) and Mathematics-Science (MS) scores. This finding is in accord with those about university students' field of study. The analysis results in the present study showed that the highest self-directed learning skills scores belonged to Fine Arts Faculty students who enter university with a talent score. The lowest mean score, on the other hand, belonged to Natural Sciences students who enter university with a Mathematics-Science (MS) score. This may be a reflection of students' high school preferences affecting their self-directed learning skills. Previous studies have also shown that students who choose their own departments, identify their learning needs and consider their preferences are more passionate about their learning and sustaining it. Özyogurtcu (2007) found in his study that students at Anatolian Fine Arts High Schools tend to more frequently choose this school on their own will and are determined to continue music education. Sarikaya and Khorshid (2009), stated that students' reasons for choosing numerical score type and studying Natural Sciences, this field has more job opportunities that offers higher income. At the same time, they also found in their study that Natural Sciences students were significantly more likely to choose their field of study upon others' recommendations and with feelings of desperation than those who choose to study Social and Health Sciences. Other studies on self-regulated learning skills, which share common dimensions with self-directed learning skills, have shown that students who enter university with a verbal score have higher self-regulated learning skills than other score types (Gömleksiz and Demiralp, 2012).

An examination of students' self-directed learning skills and their desire to pursue a graduate degree showed that students who desire to pursue graduate education had significantly higher self-directed learning skills than those who did not have a similar desire. Self-directed learners may be said to have high motivation levels, theye are keen on self-development and they aim to sustain their education. These qualities may be increasing their desire for a graduate education as a process of professional and academic development because graduate education enables individuals to advance in their fields, have in-depth knowledge in it, sustain their learning and develop themselves. In addition, individuals seeking graduate education usually appreciate learning and are motivated to learn. Saracaloglu (2008) concluded in a study that graduate students had motivation at an academically “adequate” level. Previous studies show that self-directed learning is related to attending graduate education and sustained learning (Fox, 2011; Acar, 2014; Sarmasoglu and Görgülü, 2014).

No significant difference was found between students' self-directed learning skills according to thie level of income. This may be attributed to the fact that self-directed learning is not associated with income level or financial concerns, but with the desire to learn and desire to develop oneself independently from financial concerns. Fox's (2011) study also revealed that teachers' desire to become self-directed learners was not related to financial gain. This finding may be considered as an explanation why self-directed learning skills do not vary by income level. Previous studies have also concluded that income level differentials do not affect self-directed learning skills (Atacanli, 2007; Kiliç and Sökmen, 2012; Acar, 2014).

The study finally examined the relationship between university students' self-directed learning skills and their lifelong learning tendencies. These two were found to be related with each other. A moderate and positive relationship existed between them. Lifelong learning and self-directed learning have similar properties and at times include one another. These similarities help explain the relationship between the two. While lifelong learners are defined as individuals who love to learn, who are curious and critical, they are capable of self-evaluation, they have a vision, and can choose and manage appropriate learning strategies (Candy et al., 1994), self-directed learners are defined as open, curious, organized, motivated, enthusiastic and self-controlled individuals who value learning (Jennett, 1992). Considered together, these similarities explain why lifelong learning and self-directed learning are related. In the literature too, the concepts of lifelong and self-directed learning are taken as related concepts (Brockett and Hiemstra, 1991; Greveson and Spencer; 2005; Candy, 1991 cited in Mocker and Spear, 1982; Loyens et al., 2008; Shen et al., 2014).

Our results suggest that for improved practices, instructional environments should be designed in a way to improve students' self-control skills and these environments should include the use of reflective journals, learning performance evaluation scales and cognitive and/or upper-cognitive learning strategies. In addition to these, it would be beneficial to make more space in educational settings for activities that develop students' creative thinking skills starting from elementary school. Including such activities may also help enhance academic performance at universities. For achieving higher self-directed learning skills among university students, they should be allowed to identify their own learning needs; and their opinions may be taken in to consideration when identifying learning objectives. Various learning strategies should be addressed in classes; and students should be encouraged to monitor and evaluate their own learning processes. Finally, special learning experiences which improve individuals' self-directed learning skills should be used to help them become lifelong learners. On the other hand, future qualitative studies may be conducted to explore in detail the environments in which university students acquire self-directed learning skills and/or how they advance these skills. Undergraduate and graduate students' self-regulation skills may be investigated comparatively (those in formal education vs. those in distance education; master's vs. doctoral students, etc.). Furthermore, the self-directed learning skills of academics and teachers and students from different stages of education (elementary, secondary, high school) may be explored.

## Author contributions

All authors listed have made a substantial, direct and intellectual contribution to the work, and approved it for publication.

### Conflict of interest statement

The authors declare that the research was conducted in the absence of any commercial or financial relationships that could be construed as a potential conflict of interest.
